# Twitter Response to Munich July 2016 Attack: Network Analysis of Influence

**DOI:** 10.3389/fdata.2019.00017

**Published:** 2019-06-25

**Authors:** Ivan Bermudez, Daniel Cleven, Ralucca Gera, Erik T. Kiser, Timothy Newlin, Akrati Saxena

**Affiliations:** ^1^Naval Postgraduate School, Monterey, CA, United States; ^2^Department of Computer Science, National University of Singapore, Singapore, Singapore

**Keywords:** Twitter data analysis, Munich July 2016 attack, social network analysis, meme propagation, influence spread

## Abstract

Social Media platforms in Cyberspace provide communication channels for individuals, businesses, as well as state and non-state actors (i.e., individuals and groups) to conduct messaging campaigns. What are the spheres of influence that arose around the keyword *#Munich* on Twitter following an active shooter event at a Munich shopping mall in July 2016? To answer that question in this work, we capture tweets utilizing *#Munich* beginning 1 h after the shooting was reported, and the data collection ends approximately 1 month later^1^. We construct both daily networks and a cumulative network from this data. We analyze community evolution using the standard Louvain algorithm, and how the communities change over time to study how they both encourage and discourage the effectiveness of an information messaging campaign. We conclude that the large communities observed in the early stage of the data disappear from the *#Munich* conversation within 7 days. The politically charged nature of many of these communities suggests their activity is migrated to other Twitter hashtags (i.e., conversation topics). Future analysis of Twitter activity might focus on tracking communities across topics and time.

## 1. Introduction

### 1.1. Event Background

**Figure 1:**
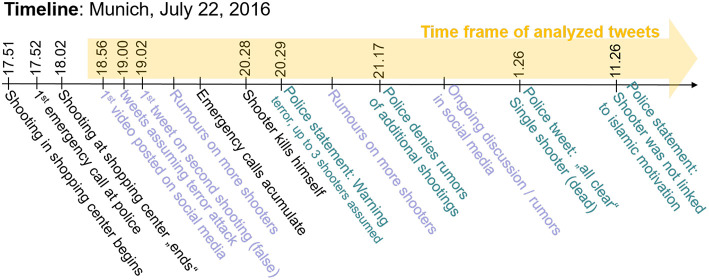
Timeline of attack (Zeitung, 2016).

On July 22, 2016, a mass shooting occurred in a shopping mall in Munich, Germany. The attacker was quickly identified by local police as an 18 year old German-Iranian dual national resident of Munich (Harrison, 2016). As is often the case after high impact incidents like mass shootings, there was a high volume of conversation in social media associated with this shooting. Conversations range from official government accounts providing instructions to affected people, speculation regarding the identity and motivation of the attacker(s), and individuals or news organizations providing reports (accurate or otherwise) of the event. As micro-blogging services like Twitter become more popular, it becomes interesting to analyze the data generated by the service in an attempt to extract topologies or trends that may provide insight into the event in question. A timeline of this event is displayed in Figure 1.

### 1.2. Motivation: Twitter Connection to Information Warfare

The Internet and Cyberspace foster many types of activities that involve different aspects of human social interaction. We can visualize and analyze the relationships that convey these interactions using network science techniques. Twitter is undoubtedly a common channel by which significant online social interaction occurs. Individuals, organizations, and nation states all use this tool as a medium of communication and many interested listeners, then retweet statements they believe deserve the attention of others. Both the real world and contrived activity generate conversations around a particular hashtag. Regardless of the authenticity of an event, the social interactions that occur during and after its occurrence have a real effect on the way humans perceive the world and can influence their future actions both in the world and in Cyberspace. To improve our appreciation for how message information both spreads and decays, we increasingly study and understand how information campaigns develop and change.

Information Warfare has been occurring for as long as parties have been trying to deceive their opponents. While the information itself may not be physical, it is considered by social scientists and the Department of Defense in Joint Publication 1 (Department of Defense, 2013) as one of the instruments of national power that the nation states utilize in order to pursue their ends. The other instruments include Diplomatic, Military, and Economic power (DIME) (Department of Defense, 2013). At first glance, Twitter seems to offer the empowerment of free speech to any user, and yet our analysis of the retweeting that occurs helps demonstrate how little many users are interested in genuine original thought. Rather, the majority of traffic tends to gravitate toward sharing the thoughts of a few accounts. We believe that such influence, while not forced by any entity, still offers tremendous power for parties engaged in Information Warfare to increase their power within the domain of Cyberspace. This power is not limited to national security and a nation's foreign policy but extends into the realms of domestic politics, sports, business, and many other areas.

Does Twitter offer the empowerment of free speech to any user? And does that make a difference? To understand this, we analyze the Twitter data we collected on the Munich attack using Netlytic (Gruzd, 2016), a software that captures data and can perform social network analysis as well. We collected dataset focused on the surge in Twitter activity using *#Munich* linked to the July 22 shootings which garnered international attention across social media and traditional reporting channels. We analyze both temporal slices of the data and the cumulative dataset to better understand how information and messages propagate across Twitter. In particular, we are interested in the community structure, its evolution, and the role of top influential leaders within these communities.

The main contributions of the paper are: (1) The collection of the hashtaged *#Munich* dataset from Twitter for an active shooter event at a Munich shopping mall in July 2016. (2) The general analysis of the cumulative network of retweets for this incident. (3) The evolution of the influence flow-based communities in temporal network of timeslices by day. In section 2 we discuss related work. Section 3 covers the problem definition and the details of the collected dataset. In sections 4 and 5, we discuss the methodology and results, respectively. The paper concludes with several future directions.

## 2. Related Work

In the current era of social networking, information sharing has been easier by posting microblogs (Kempe et al., 2003; Leskovec et al., 2007). The influence spreads very fast over the network and impacts the opinion of the users or maybe groups of users, i.e., communities (Lin et al., 2008). Researchers have studied the influence propagation on Social networking platform and their impact on network structure (Sadikov and Martinez, 2009; Chen et al., 2013; Saxena et al., 2015). Hong et al. (2011) proposed a method that successfully predicts popular tweets using the content of the message, temporal information, metadata of messages and users, structural properties of the users' social network.

Of more specific interest to us is the study of spreading behavior of tweets in case of attacks, hazards, natural calamities, etc, and how it affects the opinion of the users. Nadamoto et al. (2013) observed that the spreading of rumor during the disaster situation is different from the normal situation. In a disaster situation, the rumor goes through two or three hierarchy, but in the normal situation, it passes through many hierarchies. In the case of news spreading during disasters, Jin et al. (2014) showed that lies, half-truths, and rumors spread in the same way as true news using tweets during the Ebola crisis. On the other hand, Mendoza et al. (2010) showed that the propagation of rumor differs from the true news, and this information can be used to detect rumors using aggregated analysis on tweet dataset collected on the 2010 earthquake in Chille. Spiro et al. (2012) proposed a model for the waiting time of retweets and showed that the hazard related tweets have a shorter waiting time. For a non-disaster situations, Vosoughi et al. (2018) observed that the false news spread faster, farther and deeper, and are more prominent in the case of political news than financial, disaster, terrorism or science-related news. This research is based on Twitter data spanning 11 years comprising around 126,000 stories tweeted by around 3 million people. A brief survey on influence propagation on online social networks can be seen at Bonchi (2011).

How do communities emerge while influence spread? Gupta et al. (2016) studied the role of core-periphery structure in the information propagation to multiple communities. Complementing the spreading behavior, we are also interested in identifying influential user or users on Twitter, the emergence of influential leaders in different communities, how they shift from one community to another and how they die out (Tsur and Rappoport, 2012; Riquelme and González-Cantergiani, 2016). More specific, understanding this phenomenon based on dominant language per hashtag to trace which users overlap between the thematic and linguistic communities delineated by different information streams (Bastos et al., 2013). Our research examines several language communities that intermix with political leanings of conversations, Spanish, French, and English all use *#Munich* although it is important to remember the German discussion mostly emerged under #München. By studying the dependencies between global features such as graph topology and content features emergence helps in explaining how long members might remain in the community and the importance of repeated messaging to maintain the community of influence over time. Successful prediction of the spread of memes can improve marketing efforts whether the target is a commercial product or an idea being promoted.

Influence propagation has also been studied using the multilayered structure of online social networks. The layers depict either different type of relationship, allowing the researchers to perform studies at different granularity (Li et al., 2012; Zhuang and Yağan, 2016) or the layers representing followers, mentions or retweeting (Borondo et al., 2015). We also include the multilayer aspect in our research in a different way, namely temporally. Wang et al. (2008) stated that most nodes lack stability in the evolution of the network between time steps, and the manner in which time is partitioned will determine how communities are detected. This inspires our analysis to examine if and how accounts migrate between communities over time. Yet, in terms of stability, Romero et al. (2011) highlighted that hashtags on politically controversial topics are particularly persistent, with repeated exposures continuing to have unusually large marginal effects on adoption. In this research, we do not specifically examine how long certain messages persist, but the observation about political messages lasting longer is related to how long individuals choose to continue retweeting the same leader accounts over multiple days. That information is captured in the multipartite temporal network, and it is shown in section 5.

Smith et al. (2014) from the Pew Research Center found six different network structures (Polarized Crowds, Tight Crowd, Brand Clusters, Community Clusters, Broadcast Network, Support Network) that emerge in social media networks. They study how the structures differ based on the content of the issues driving the discussion, highlighting the importance that most real social networks are usually a hybrid of multiple structures. The research shows that Broadcast Network, and Support Network have large size groups, Tight Crowd has medium size groups, and Brand clusters and Community Clusters have many small sized groups. The structures of interest to the *#Munich* dataset were the Community Clusters, and Broadcast Network. Both of these structures appeared within the context of the retweets in the month following the July 2016 attack in Munich. The Pew researchers give voice to the idea that mapping the social landscape using networks helps interpret trends, topics, and implications of the technologies being used. Our analysis of the *#Munich* data regarding the polarized crowd supports the Pew team's statement that if a topic is political, it is common to see two separate groups take shape and they form two distinct discussion groups that mostly do not interact with each other. The groups are recognizably liberal or conservative (Smith et al., 2014). Each group links to a different set of influential people or organizations that can be found at the center of each conversation cluster.

## 3. Problem Definition and Dataset

The broader problem examined in this research is how social media spheres of influence in Cyberspace can be employed to conduct information operations campaigns. We analyze the communities of influence in the considered dataset and their evolution over time.

The approach to solving this problem uses efforts similar to Pew Research (Smith et al., 2014) work on Social Media, personalized for the *#Munich* Dataset. Our background research leads to the understanding that the structure of the network we create affects how communities emerge. In this work, we focus on retweets only, because they convey the aspect of influence, as individuals choose to associate with particular leader's thoughts. The network's nodes are thus the Twitter accounts that have retweets at least once, and directed edges connect retweeting accounts to the account of origin for that message.

The original data captured in Netlytics consists of 13 files of total 655 MB (Gruzd, 2016). It conveys all Tweets captured from July 22, 2016 to August 22, 2016 labeled with *#Munich*. This discussion topic involved 147,116 Twitter accounts that either tweeted or re-tweeted *#Munich* messages during those 32 days. Each row of the dataset containes several categories including the text of the Tweet, date, time, author, type of device it was posted from when user/account was created, and the Twitter profile location.

Our research focuses on the Tweets that contain the retweet indicator, “RT@”, in the text or body of the message. Of the total 925,019 Tweets, 79.8% were retweets, and 72% of all retweets occurred between July 22 and July 25 which corresponds to the first 3 days after the shooting. The Tweets cover several languages including English, French, and Spanish, all of which use the spelling Munich for the city. However, very few German language Tweets are captured because German Twitter users use the German spelling of “München” instead of “Munich.”

## 4. Methodology

The raw data was used to build a directed graph *G*_0_ of the *#Munich* data using the following methodology.

(1) Every unique Twitter account that occurred in our data is represented by a node,
(2) A directed edge is placed from node *u* to node *v* if user *u* retweets the tweet that was initially posted by user *v*,
(3) Edge weights represent the number of times user *u* retweeted user *v*'s tweets, and
(*) Edges in *G*_0_ did not contain any temporal information.

The resulted graph *G*_0_ has 147, 116 nodes, 191, 002 edges. To this graph we apply a standard community detection algorithm called Louvain (Blondel et al., 2008). The algorithm assigns nodes randomly to communities, measures the strength of the community partition using modularity (Newman, 2006), and shuffles neighbors from one community to another while maximizing modularity. The result of the Louvain algorithm is 5, 807 communities, which will become part of our cumulative analysis of this network.

**Figure 2:**
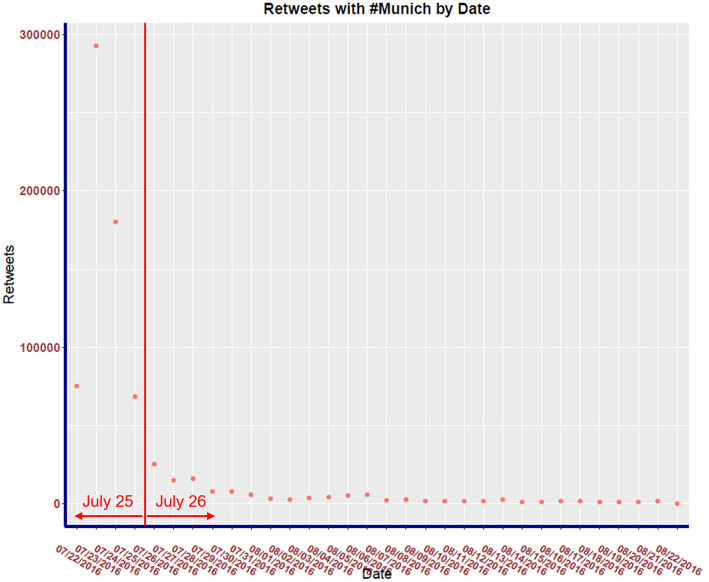
*#Munich* retweets by date.

Our temporal analysis of the raw data reveals that over 72% of all retweets occurred between July 22, 2016 and July 25, 2016 as shown in Figure 2. For temporal analysis, we thus focus the analysis on these 4 days, for which we build sub-graphs *G*_22_, *G*_23_, *G*_24_, *G*_25_ using the same methodology described above, but only capturing retweets of the top twenty leaders for each day.

Building upon Smith's observations (Smith et al., 2014), we propose and compute the leader score for every node. This score shows which user accounts are influential within a community, and provide a relative scale of their influence. The leader score for every node *u* in *G* is computed as follows.

(1) $$\begin{array}{r} leaderscore\left(u\right)=\frac{deg_{in}\left(u\right)}{deg_{out}\left(u\right)+1}. \end{array}$$

We further propose a metric to compare leader-centric communities across time, computed in two steps: (1) run Louvain community detection on sub-graphs *G*_22_, *G*_23_, *G*_24_, *G*_25_, and then (2) add an edge between communities if they have a shared user. Let *U*_*t*_ be the set of users comprising community *U* on day (*t*), and *V*_*t*+1_ be the set of users comprising community *V* on day (*t* + 1). We then compute the similarity between two communities as,

(2) $$\begin{array}{r} similarity(U_{t},V_{t+1})=average\left(\frac{U_{t}\cap V_{t+1}}{U_{t}},\frac{U_{t}\cap V_{t+1}}{V_{t+1}}\right). \end{array}$$

Similarity metric assigns the value while considering the sizes of the communities, as community sizes may vary a lot due to their sphere of influence. Next, we present the analysis results using the discussed metrics.

## 5. Results and Analysis

In exploring the data set of all the Tweets with *#Munich*, we notice that about 80% of the Tweets were individuals retweeting other users. This dynamically captures the influence of a very small portion of the overall accounts, because these tweets include content that a large number of other users identify with as they get retweeted.

The distribution of retweets vs. day is shown in Figure 2. Observe that the distribution of all retweets for 32 days has a strong positive skew with the majority of retweets occurring the day after the attack. Notice that within a week, the activity returns to a level similar to before the attack.

**Figure 3:**
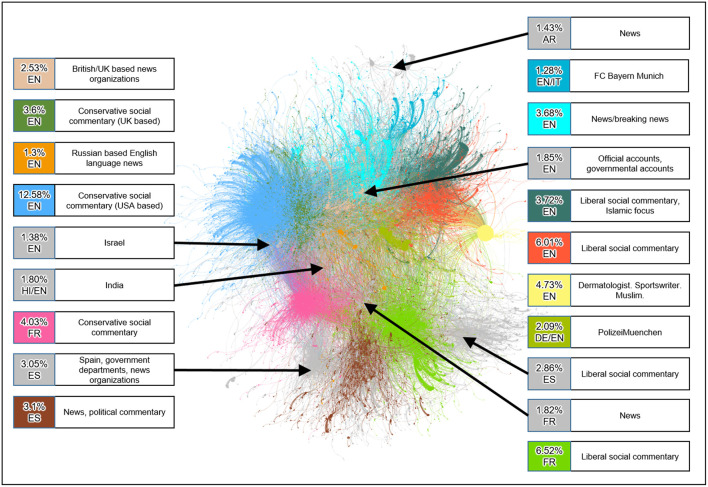
Visualization of G*.

We begin our study with the community structure of the cumulative dataset using Louvain algorithm, identifying 5, 807 communities. For better visualization, we create a graph G^*^ from *G*_0_ by selecting the 20 largest communities in *G*_0_. G^*^ contains less than 1% of the communities, but it still accounts for over 70% of the nodes and 75% of the edges in *G*_0_. Figure 3 shows a plot of the G^*^ using the ForceAtlas visualization from Gephi (Bastian, 2009). A large number of edges or high edge weights between two communities corresponds to greater proximity on the visualization; whereas communities which share few or no edges will be spaced further apart on the visualization.

The information box for each community in Figure 3 conveys the following information:

1. The percent of nodes in *G*_0_ that the community comprises.
2. The predominant language of the community, as,
  - EN: English
  - ES: Spanish
  - FR: French
  - HI: Hindi
3. A brief characterization of the community based on the profiles of its leaders using commonly accepted definitions of conservative and liberal social views. The term social commentary is used to emphasize the proffering of opinions rather than the objective conveyance of information.

Figure 3 reveals a partitioning of the communities along linguistic and political lines. We observe that a community built around a common language and/or shared political views is more likely to have a higher edges density. One can visually interpret the data in Figure 3 as follows:

- Horizontally (left to right): socially conservative communities, politically neutral communities/news sources, socially liberal communities
- Vertically (top to bottom): English language communities, French language communities, Spanish language communities.

Note that the FC Bayern Munich community might be outside the scope of the study of the July 22nd attack, rather tweets on football using the same hashtag(#Munich). Since the data captured it anyway, we have shown it in the analysis.

The partitioning of communities along language and political views reinforces the findings of the Pew study (Smith et al., 2014). The relatively small size of the communities represents news sources given a large number of Twitter followers many of these news outlets have. This is likely a result of how the network is built since it only captures the accounts who actively retweet others yet fail to capture passive users who consume Tweets but do not actively retweet.

Terrorist events such as the Munich attack create a unique circumstance where we assume that leaders within preexisting communities (fundamental communities) attach themselves to a particular hashtag (e.g., topic) and form topic communities. This creates the following cases for followers and leaders.

1. A user exists in the fundamental community but not in the topic community
2. A user exists in both the fundamental community and the topic community
3. A user exists in a topic-specific community but not in the underlying fundamental community.

For example, user *u* agrees with the sentiment of leader *v*'s Tweet on the topic of the *#Munich* and retweets *v*'s message. Users *u* and *v* are in the same topic community, but not necessarily in the same fundamental communities if in general their views do not coincide.

**Figure 4:**
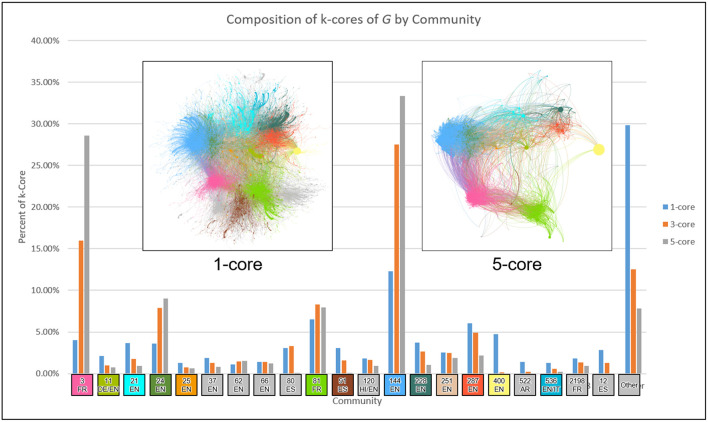
Community partition within different k-cores.

To understand the influential hierarchy of the network, we first apply the core-periphery analysis of the network using K-shell decomposition method (Seidman, 1983). The k-shell decomposition method assigns a k-shell value to each node, and it works in the following way. The k-shell method first removes all nodes of degree one until there is no node of degree one or less, and assigns them k-shell value 1. Iteratively, it will remove nodes of degree 2, 3, 4…and will assign them k-shell value 2, 3, 4…respectively. While removing the nodes of degree *k*, if any node is ended up having degree *k* or less, will also be removed in the same iteration. The method is stopped once each node has been assigned a k-shell value. *k*-core of a network contains all the nodes having k-shell value equal to or higher than *k*. Figure 4 presents the split of the 1-core, 3-core and the 5-core between the communities for a better understanding of the core-periphery structure. We observe that the cores of different communities are connected with each other, thus leaders communicate or influence each others. We also observe that the smaller communities do not have higher influential nodes having a higher k-shell value, which could be the reason why they did not become larger communities overtime. Next, we do the temporal analysis of retweet networks for better understanding the role and evolution of the communities.

### 5.1. Temporal Leader Networks

**Figure 5:**
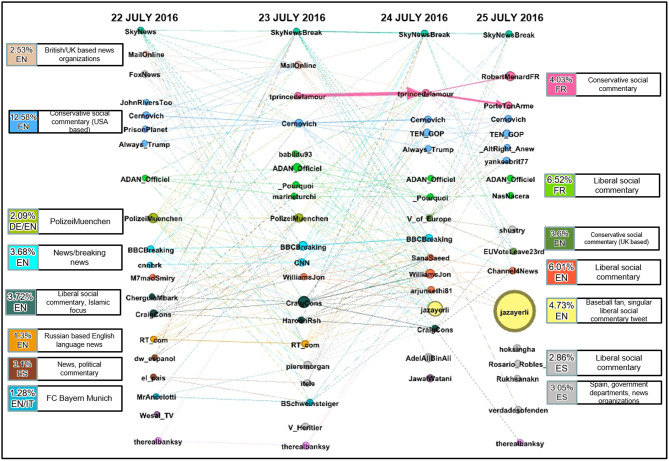
Multipartite temporal community daily evolution.

In this section, we study the evolution of the communities found in the cumulative graph for the first 4 days succeeding the attack. This subset is deemed adequate by examining the frequency of retweets in each day for the whole period. As seen in Figure 2, the majority of traffic occurs from the 22 to 25 of July 2016. This subset of data is used to create the multilayer network seen in Figure 5.

In this multilayer network, the nodes represent each of the top 20 communities, and a single layer is created for each day. The nodes are sized by the number of users in that community and colored by the communities they belong to in the cumulative graph. The label of each node corresponds to the leader in that community as defined by Equation (1). We then add an edge from a node in one layer to a node in another layer if there are any shared users between the two communities. This captures the continuity of community membership. The weight of the edge is then computed using Equation (2) as described in the methodology.

The resulting network provides significant insight into the evolution of communities over time. Figure 5 provides visualization for the migration of users and leaders between different communities. We also observe that community leaders appear or disappear each day depending on whether they generate a tweet message and the volume of retweets. For example, the Russian based English language news (RTcom) community dies out after July 23.

We further observe that a significant amount of users retweet from the same community. Although this observation is prevalent in the data, it is most evident in the community labeled as French conservative social commentary. We observe that a high amount of users that retweeted from @tprincedelamour on July 23, 2016, did so again on the next day.

Following communities from left to right we see how they can merge from several nodes to one or split from one over each day, as is the case with the English conservative social commentary (USA based) community. Lastly, the @jazayerli community is seen to grow from the 24 to 25 of July with no connecting edge. The lack of an edge between these two nodes is because the community consists of just one Twitter message generated by @jazayerli that is retweeted several times over the 2 days.

The cumulative and temporal graphs of the Twitter data complement each other by providing overlapping insight into the communities described. Figure 5 provides insight into the nature of each community; how did leaders and followers' activities for a given community change across time. Figure 3 provides an overview of each community and its relative importance across time and the degree to which communities and leaders are connected. Examining the yellow colored community lead by @jazayerli, it becomes clear from Figure 3 that this community is a peninsula (a very small community) and Figure 5 illustrates that this particular community dominated the *#Munich* retweets on July 25.

## 6. Conclusions and Further Directions

In this work, we collect and analyze the Twitter data of *#Munich* July 2016 attack corresponding to a month-long period after the July 22 shootings in Munich. We study the community structure in the cumulative dataset as well as daily partitions and classify each community based on the nature of its leaders and their tweets. This study provides insight on how information spreads on Twitter in case of an event, and we observe how the important leaders disappear from the network of *#Munich* retweets after a week of the attack. The leaders in the first week tended to be news organizations or social leaders with strong or extreme views. Communities expressing strong opinions were the most active; however, as mentioned, the collected data is unable to account for passive users (e.g., users who may read a Tweet and internalize the information or message but do not retweet the message). One can further study the impact of the event on other users who are not directly involved in tweeting and retweeting, however, have been affected by the event. The analysis can also be extended to different social media platforms for better understanding.

This research opens up several questions to be studied for a better understanding of the evolution of the network in case of terrorist attacks. One can identify the leaders and follow them across multiple hashtags to determine topic communities of leaders for each hashtag. By comparing a leader's topic networks and identifying users that retweet the leader across multiple different topics, we can understand the development of the fundamental and topic-based communities represented by that leader. These communities can be further classified based on different parameters, such as is the community passive where the leader has many followers, but few retweets; or is it active where the majority of users following a leader actively retweet the leader across many different topics.

All these approaches will be fruitful in a deeper understanding of how communities generate influence in social media networks. Given the increasing use of online social media, the implications for how corporations, organizations, and nation states conduct influence campaigns will continue to grow as part of future information operations.

## Data Availability

The datasets generated for this study are available on request to the corresponding author.

## Author Contributions

IB, DC, EK, and TN collected the dataset, discussed the research methodology, implemented the project, and contributed to the writing of the manuscript. RG and AS contributed to the research methodology and its implementation, analysis of the results, and writing of the manuscript.

### Conflict of Interest Statement

The authors declare that the research was conducted in the absence of any commercial or financial relationships that could be construed as a potential conflict of interest.
